# Prognostic value of ^68^Ga-DOTATATE PET/CT in assessing cardiac involvement in autoimmune diseases: a prospective study

**DOI:** 10.3389/fcvm.2025.1598638

**Published:** 2025-10-10

**Authors:** Xue Lin, Na Niu, Wei Chen, Wuwan Wang, Ximin Shi, DaChun Zhao, XiaoHang Liu, Fanglu Wang, Ligang Fang, Li Huo

**Affiliations:** ^1^ Department of Cardiology, State Key Laboratory of Complex Severe and Rare Diseases, Peking Union Medical College Hospital, Chinese Academy of Medical Science and Peking Union Medical College, Beijing, China; ^2^ Department of Nuclear Medicine, Peking Union Medical College Hospital, Chinese Academy of Medical Science and Peking Union Medical College, Beijing, China; ^3^ Department of Pathology, Peking Union Medical College Hospital, Chinese Academy of Medical Science and Peking Union Medical College, Beijing, China

**Keywords:** ^68^Ga-DOTATATE PET/CT, myocardial macrophage infiltration, autoimmune diseases, prognostic value, clinical outcomes

## Abstract

**Background:**

This prospective study aimed to evaluate the prognostic value of ^68^Ga-DOTATATE PET/CT imaging in assessing myocardial macrophage infiltration in patients with autoimmune diseases and its relationship with clinical outcomes, specifically all-cause mortality and cardiovascular rehospitalization.

**Methods:**

A total of 36 patients with cardiac involvement due to autoimmune diseases were enrolled. All underwent ^68^Ga-DOTATATE PET/CT imaging to assess left ventricular mononuclear macrophage infiltration. Clinical data, including myocardial biopsy results and left ventricular SUV values (LVmax and LVmean), were recorded. Prognostic thresholds were identified using ROC curve analysis, while Kaplan–Meier survival were used to analyze the relationship between myocardial macrophage activity and clinical outcomes.

**Results:**

Of the 36 patients, 9 died and 9 were rehospitalized for cardiovascular reasons. ROC analysis demonstrated that LVmax ≥ 2.405 predicted all-cause mortality with an AUC of 0.96 (95% CI: 0.92–1.00, *P* < 0.0001), while LVmean ≥ 1.36 predicted cardiovascular rehospitalization with an AUC of 0.87 (95% CI: 0.79–0.96, *P* < 0.0001). High ^68^Ga-DOTATATE uptake thus significantly correlated with adverse clinical outcomes, surpassing traditional markers such as ejection fraction and inflammatory biomarkers. Moreover, ^68^Ga-DOTATATE imaging results were more consistent with the patients’ clinical conditions compared to myocardial biopsy, highlighting its superior diagnostic utility in assessing diffuse myocardial inflammation.

**Conclusion:**

^68^Ga-DOTATATE PET/CT imaging offers a valuable, non-invasive, and quantitative method for assessing myocardial inflammation in autoimmune diseases. This technique shows potential for improving personalized treatment and prognosis by identifying patients at higher risk for adverse outcomes in autoimmune diseases.

## Background

Autoimmune diseases can profoundly compromise cardiac health by triggering severe inflammatory responses (1). These responses can damage various components of the heart, including the pericardium, myocardium, conduction system, valves, coronary arteries, and microcirculation, often resulting in devastating outcomes (2–4). The diagnosis and treatment of cardiac involvement in autoimmune diseases typically require collaboration between cardiologists and immunologists. Cardiac damage is typically managed through interventions such as coronary procedures, pacemaker implantation, and heart failure management, while immunological treatment often includes steroids, immunosuppressants, and biologics, guided by inflammatory markers and clinical presentation. Despite these comprehensive approaches, many patients still face high rates of cardiac-related mortality, primarily due to the lack of quantitative, non-invasive methods to assess myocardial inflammation, which limits clinicians’ ability to tailor treatments and prevent adverse outcomes (5).

Although the molecular mechanisms of autoimmune diseases differ, their cardiac involvement shares common pathophysiological features, including chronic inflammation, cytokine dysregulation, and elevated cardiovascular risk (6, 7). A key mediator in these processes is macrophage infiltration, particularly pro-inflammatory M1 macrophages, which are involved in the progression and prognosis of autoimmune diseases like systemic lupus erythematosus, vasculitis, rheumatoid arthritis, myositis, and Sjögren's syndrome etc (8–12). Chronic activation of macrophages results in cardiac damage, fibrosis, and remodeling, ultimately impairing heart function (7, 13–15). Recent studies have shown that targeting macrophage functions may improve outcomes in immune-mediated diseases (16, 17). The similar pathophysiological changes in myocardial involvement across various autoimmune diseases provide a foundation for using non-invasive methods to assess macrophage-driven inflammation, thus improving diagnosis, guiding treatments, and preventing adverse outcomes.

It has been shown that activated macrophages overexpress somatostatin receptor subtype−2 (SSTR2) (18). Gallium-68-DOTA-0-Tyr3-Octreotate (^68^Ga-DOTATATE), a PET tracer that binds to SSTR2, was initially used to image neuroendocrine tumors clinically (19). Recently, this imaging agent has been validated as a marker of proinflammatory M1 macrophages in vulnerable atherosclerotic plaques (20, 21) and residual post-infarction myocardial inflammation (22). Additionally, ^68^Ga-DOTATATE does not show significant uptake in normal myocardial tissue, yet demonstrates increased accumulation correlated with various cardiac disease conditions (23). These findings suggest that ^68^Ga-DOTATATE imaging could serve as a non-invasive method for assessing macrophage infiltration and chronic inflammation in the heart, offering valuable insights into the progression of immune-mediated diseases and their impact on cardiac outcomes.

These findings support the potential of ^68^Ga-DOTATATE imaging as a non-invasive method for evaluating macrophage infiltration and chronic myocardial inflammation, providing valuable insights into the progression of immune-mediated cardiac diseases. However, its clinical utility specifically in autoimmune-associated myocardial inflammation remains uncertain.

This study uses ^68^Ga-DOTATATE imaging to assess myocardial involvement in patients with various autoimmune diseases. It evaluates the prognostic impact of macrophage inflammation on cardiac outcomes, using all-cause mortality and cardiovascular rehospitalization as key endpoints. The aim is to address the current gap in effective non-invasive methods for assessing myocardial inflammation, with the potential to establish diagnostic thresholds for more precise treatment of cardiac involvement in autoimmune diseases.

## Methods

### Study design and ethics approval

**Figure 1:**
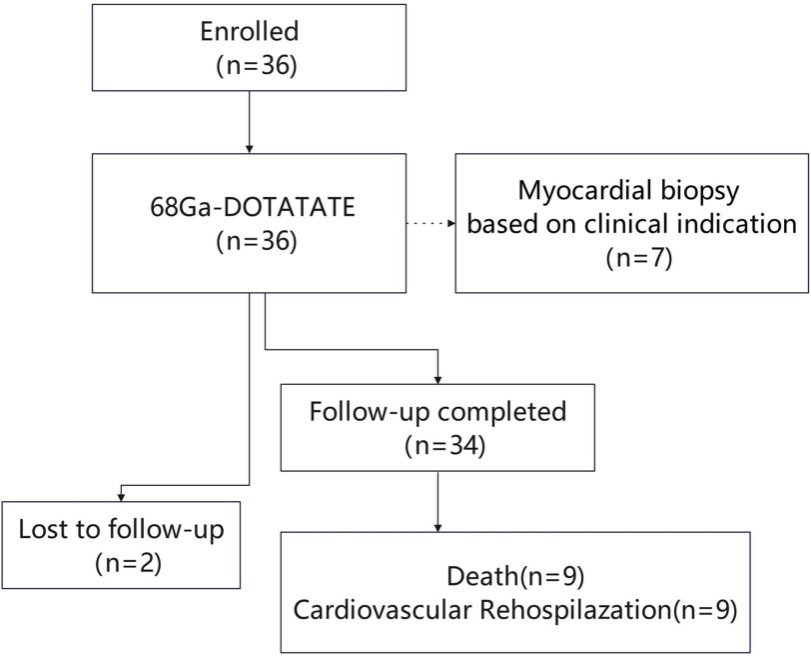
Study enrollment flowchart illustrating patient inclusion, examinations performed, follow-up completion, and clinical outcomes.

This single-center, prospective observational study was conducted at Peking Union Medical College Hospital from March 1, 2019, to October 22, 2022. Patients diagnosed with cardiac involvement due to autoimmune diseases were enrolled. Inclusion criteria included a confirmed diagnosis of an immune-mediated disease with cardiac involvement, presenting with clinical symptoms (e.g., shortness of breath, chest pain, palpitations) along with elevated cardiac enzymes, abnormal ECG, or cardiac imaging indicating structural or functional abnormalities. These abnormalities were confirmed to be related to the systemic immune disease by both immunologists and cardiologists. Patients were required to have received standardized treatment, including steroid therapy, immunosuppressants, biologics, and any necessary cardiac interventions. The study enrollment flowchart is shown in Figure 1. Generally, patients with Behçet's disease are primarily treated with cyclophosphamide (CTX), leflunomide (LEF), or azathioprine (AZA). Patients with myositis typically receive a combination of cyclophosphamide (CTX) and methotrexate (MTX) or cyclophosphamide (CTX) with cyclosporine A (CSA). Those with vasculitis are mainly treated with cyclophosphamide (CTX), methotrexate (MTX), and tocilizumab (TCZ). Additionally, individuals with systemic lupus erythematosus (SLE) usually undergo treatment with hydroxychloroquine (HCQ) combined with either leflunomide (LEF) or mycophenolate mofetil (MMF), or hydroxychloroquine (HCQ) paired with tacrolimus (TAC) and mycophenolate mofetil (MMF).

Regular follow-up visits were mandatory for treatment adjustments. Exclusion criteria included cardiac neuroendocrine tumors and severe multi-system failure that precluded effective follow-up. The study was approved by the ethics committee of Peking Union Medical College Hospital (IRB protocol #JS-2452), and all participants provided written informed consent.

### Baseline characteristics and echocardiography

Clinical and demographic characteristics were collected at enrollment through chart reviews, laboratory data, and auxiliary examinations. Baseline data included sex, age, BMI, blood pressure, heart rate, laboratory parameters, and echocardiographic measurements. eGFR was calculated using the CKD-EPI equation (24). Transthoracic echocardiography was performed with commercially available equipment (Vivid E9, GE Medical Systems, Horten, Norway). Left ventricle systolic function was evaluated using Biplane Simpson's Ejection Fraction (Biplane LVEF). Right ventricle systolic function was assessed using Tricuspid Annular Plane Systolic Excursion (TAPSE). Cardiac remodeling was primarily evaluated using LV mass index (LVMI) or relative wall thickness (RWT) (25).

### ^68^Ga-DOTATATE PET/CT

All patients underwent ^68^Ga-DOTATATE PET/CT scans at enrollment. No dietary restrictions or fasting were required. Patients on long-acting somatostatin analogues discontinued these medications at least four weeks before examination (26). PET/CT imaging began approximately 45 minutes after intravenous injection of ^68^Ga-DOTATATE (3.3 ± 0.8 mCi). One bed position covering the thoracic region (heart centered) was acquired in 3D mode for 20 minutes using a time-of-flight PET/CT scanner (Polestar m660, SinoUnion Healthcare Inc., China). CT scanning parameters included a tube voltage of 120 kV, tube current-time product of 160 mAs, pitch of 1.3, slice thickness of 2.5 mm, and rotation time of 0.5 s. PET images were reconstructed using an ordered subset expectation maximization (OSEM) algorithm (2 iterations, 10 subsets, Gaussian filter of 4.5 mm in full width at half maximum, 256 × 256 matrix), with corrections for attenuation (CT-based), dead time, random events, and scatter.

Analysis of myocardial ^68^Ga-DOTATATE uptake was performed using MIM software. Regions of interest (ROIs) were manually delineated in three dimensions (3D), encompassing the entire left ventricular myocardium with approximately 10 mm thickness from base to apex on each PET/CT fused slice. LVmax was defined as the highest SUV among all voxels within the myocardial ROI, representing peak myocardial inflammation. LVmean was calculated as the average SUV of all voxels within the myocardial ROI. Given the potential dilution of focal areas of intense uptake by SUVmean, LVmax was selected as the primary prognostic metric for its robustness and minimal sensitivity to ROI delineation variability.

### Myocardial biopsy and myocardial pathology

Among the enrolled patients, seven underwent myocardial biopsy, specifically targeting the mid-segment of the interventricular septum on the right ventricular surface. The procedure was performed under sterile conditions. After establishing venous access, typically through the jugular or femoral vein, a guiding sheath was advanced into the right ventricle under fluoroscopic guidance. Argon myocardial biopsy forceps (Argon Medical Devices, Plano, TX, USA) were introduced through the sheath and directed to the target area on the right ventricular surface of the interventricular septum. Three to five biopsy samples were routinely obtained to ensure sufficient tissue while minimizing trauma. The procedure was closely monitored for immediate complications, and the collected samples were sent for histopathological examination (27). Immunohistochemical staining using anti-CD68 antibody was performed on myocardial specimens to identify macrophages.

### Follow-up and outcome measures

Patients received standard medication therapy and were regularly followed up at the cardiac outpatient clinic. Information on their current status, medication use, and any necessary re-examinations was collected through routine clinical visits or telephone calls. In July 2024, a final follow-up was conducted, with the primary endpoints being all-cause mortality and cardiovascular rehospitalization.

### Statistical analysis

Statistical analyses were performed using SPSS (Version 23; IBM Corp., Armonk, NY, USA). The normality of continuous data was assessed using one-sample Kolmogorov–Smirnov tests and histograms. Continuous variables were expressed as mean ± SD for normally distributed data or median (IQR) for non-normally distributed data. Levene's test evaluated the homogeneity of variances. Normally distributed variables were compared using an unpaired *t*-test (for homoscedastic data) or Welch's correction (for non-homoscedastic data). Non-normally distributed variables were compared using the Mann–Whitney *U*-test. Categorical variables were expressed as percentages and compared using Pearson's *χ*² test or Fisher's exact test.

Receiver operating characteristic (ROC) curve analysis was conducted to determine area under the curve (AUC) for myocardial ^68^Ga-DOTATATE uptake (SUV LVmax and SUV LVmean) concerning two different outcomes (all-cause mortality and cardiovascular rehospitalization). The optimal threshold values were identified based on the Youden index, with sensitivity and specificity explicitly reported. Kaplan–Meier survival analysis was then performed using these identified threshold identified from the ROC curve, and differences between groups were assessed by the log-rank test. Given the exploratory nature and relatively small sample size of this study, we did not apply corrections for multiple comparisons. Thus, the results should be interpreted with caution due to the potential increase in Type I errors.

Multivariate binary logistic regression analysis was conducted as an exploratory analysis to assess whether myocardial LVmax and LVmean independently predicted adverse clinical outcomes after adjusting for relevant clinical covariates. Due to the limited sample size, logistic regression was chosen instead of Cox regression analysis. The detailed results of this analysis are presented in the Supplementary Material (Supplementary Table S1). These exploratory findings should be interpreted cautiously and validated in future larger-scale studies. A two-tailed *P*-value of <0.05 was considered statistically significant.

## Results

### Baseline characteristics and outcomes

A total of 36 patients with cardiac involvement due to autoimmune diseases [17 men, 19 women; median age: 41 (29–64) years] were enrolled in the study. Patients were enrolled at a median interval of 2.8 months after their autoimmune disease diagnosis (range: 0.1–226 months; IQR: 0.3–33 months).The cohort included 11 patients with vasculitis [including 2 with eosinophilic granulomatosis with polyangiitis (EGPA), 3 patients with Takayasu arteritis, 6 patients with systemic vasculitis], 8 with myositis, 4 with systemic lupus erythematosus (SLE), 4 with autoimmune myocarditis, 4 with Behçet's disease, 2 with systemic sclerosis, 2 with undifferentiated connective tissue disease, and 1 with Sjögren's syndrome. The follow-up period ranged from 0.5 to 65 months, with a mean follow-up time of 44 (20–58) months. Two patients were lost to follow-up; they discontinued participation and could not be reached, leaving their survival status unknown.

Baseline clinical data for the study population, grouped by patients who experienced adverse outcomes (death or cardiovascular-related rehospitalization) and those who did not, are summarized in Table 1. During the study, 9 patients died: 4 from cardiogenic shock, 3 from sudden cardiac death, 1 from respiratory failure due to alveolar hemorrhage, and 1 from cerebral infarction. The clinical data of these deceased patients are detailed in Table 2. The median survival time for patients who died was 9 [3.5–13.8] months. Additionally, 9 patients were readmitted for cardiovascular reasons, with a mean time to cardiovascular readmission was 37 ± 21 m, and 12 patients experienced composite endpoints (death or cardiovascular-related rehospitalization). Depending on the condition, the duration of immunosuppressant use is 46 [13, 65] months.

**Table 1:** Baseline characteristics in patients With and without death or cardiovascular rehospitalization.

Variable	Survivor (*n* = 25)	Death (*n* = 9)	*P* value	Non-rehospitalization (*n* = 25)	Rehospitalization (*n* = 9)	*P* value
Sex (Male)	13 (52)	3 (33.3)	0.458	13 (52)	3 (33.3)	0.448
Age (year)	38 (28, 51)	64 (40, 71)	0.239	39 (29, 64)	47 (34, 67)	0.345
BMI (kg/m^2^)	22.83 ± 4.13	23.42 ± 4.49	0.736	22.00 ± 4.03	25.50 ± 3.69	0.03
SBP (mmHg)	117 ± 20	111 ± 27	0.50	118 ± 21	108 ± 25	0.284
DBP (mmHg)	69 ± 15	70 ± 18	0.874	70 ± 16	67 ± 15	0.59
HR (bpm)	77 (72, 90)	92 (82, 101)	0.03	83 (75, 95)	80 (73, 92)	0.716
cTnI (μg/L)	0.024 (0.0125, 0.133)	0.242 (0.045, 1.187)	0.036	0.046 (0.017, 0.251)	0.033 (0.017,0.724)	0.953
CK (U/L)	45 (35, 108)	40 (25, 59)	0.495	52 (38, 108)	36 (19, 64)	0.07
CK-MB (μg/L)	1.1 (0.5, 2.6)	2.6 (2.025, 4.275)	0.07	2.0 (0.5, 3.6)	1.65 (0.73, 2.85)	0.818
NT-proBNP (pg/ml)	562 (196, 3,597)	3,456 (1,979, 6,073)	0.03	861 (285, 3,633)	2,691 (526, 5,525)	0.31
Alb (g/L)	38 (36, 46)	37 (35, 38)	0.15	38 (35, 42)	37 (36, 42)	0.91
LDH (U/L)	216 (198, 336)	344 (223, 383)	0.21	267 (209, 359)	212 (165, 384)	0.42
Hb (g/L)	128 ± 22	111 ± 22	0.05	123 ± 25	127 ± 19	0.63
Cr (μmol/L)	70 (57, 91)	91 (66, 198)	0.40	76 (57, 97)	74 (63, 94)	0.94
eGFR (ml/min/1.73 m^2^)	97 ± 32	68 ± 40	0.04	89 ± 40	89 ± 24	0.98
hsCRP (mg/L)	2.87 (0.88, 17.05)	1.49 (0.63, 26.90)	0.98	4.2 (1.02, 17.05)	0.97 (0.38, 26.50)	0.32
Echocardiographic parameters						
LA (mm)	37 ± 6	40 ± 6	0.12	36 ± 7	41 ± 4	0.06
LVEDD (mm)	55 (48, 60)	53 (47, 56)	0.47	54 (48, 59)	56 (54, 62)	0.20
LVESD (mm)	42 ± 10	39 ± 7	0.42	40 ± 9	44 ± 8	0.21
IVS (mm)	8 (7, 9)	8 (7.5, 11)	0.22	8 (7, 9.5)	8 (7, 11)	0.94
PWT (mm)	8 (6, 9)	9 (7.5, 9.5)	0.334	8 (7, 9.5)	8 (6, 9)	0.42
TAPSE (mm)	17 ± 5	17 ± 4	0.93	16 ± 5	18 ± 4	0.34
TR (m/s)	2.4 (2.2, 2.9)	2.4 (2.2, 3.2)	0.97	2.4 (2.2, 2.9)	2.2 (2.1, 3.3)	0.49
PASP (mmHg)	33 (26, 50)	34 (30, 59)	0.57	32 (26, 44)	49 (32, 65)	0.25
LVMI (g/m^2^)	98.37 ± 28.43	104.68 ± 32.30	0.59	100.07 ± 31.89	100.53 ± 22.98	0.97
Biplane EF%	46 ± 15	50 ± 14	0.57	49 ± 15	43 ± 15	0.39
RWT	0.29 (0.25, 0.33)	0.32 (0.29, 0.37)	0.25	0.31 (0.26, 0.34)	0.27 (0.22, 0.32)	0.23
^68^Ga-DOTATATE uptake						
LVmean	1.02 ± 0.36	1.31 ± 0.22	0.03	1.01 ± 0.35	1.33 ± 0.26	0.02
LVmax	1.96 ± 0.70	2.51 ± 0.34	0.03	1.95 ± 0.67	2.53 ± 0.48	0.02
TBRmax	3.87 ± 1.35	4.23 ± 1.19	0.49	3.71 ± 1.29	4.69 ± 1.11	0.05
TBRmean	2.54 ± 0.79	2.66 ± 0.72	0.71	2.42 ± 0.75	3.00 ± 0.65	0.05
Medication						
*β*-blockers	16 (64)	7 (78)	0.45	16 (64)	7 (78)	0.68
ACEi/ARB/ARNI	15 (60)	3 (33)	0.25	12 (48)	6 (67)	0.45
Spirolactone	15 (60)	4 (44)	0.42	13 (52)	6 (67)	0.70
Diuretics	14 (56)	8 (89)	0.11	14 (56)	8 (89)	0.11
Cardiotonics	2 (8)	4 (44)	0.03	3 (25)	3 (33)	0.31
CCB	3 (25)	2 (22)	0.59	5 (20)	0 (0)	0.29
Glucocorticoids/immunosuppressive agents	19 (76)	7 (78)	1.00	19 (76)	7 (78)	1.00
Anti-coagulation	6 (24)	3 (33)	0.67	4 (16)	5 (56)	0.03

Data are shown as mean ± SD or median (IQR) for continuous outcomes and *n* (%) for categorical outcomes. *P* values were based on unpaired t-test or Mann–Whitney test for continuous outcomes and Pearson Chi-squared test or Fisher exact test for categorical outcomes.

ACEi, Angiotensin-Converting Enzyme inhibitors; Alb, albumin; ARB, Angiotensin receptor blockers; ARNI, angiotensin receptor neprilysin inhibitor; Spironolactone, aldosterone receptor antagonist; Cardiotonics, cardiac glycosides or positive inotropic agents; CCB, calcium channel blocker; BMI, body mass index; Biplane EF%, Biplane Simpson's Ejection Fraction; CK, creatine kinase; CK-MB, creatine kinase-MB; Cr, creatinine; hsCRP, high-sensitivity C-reactive protein; cTnI, cardiac troponin I; DBP, diastolic blood pressure; eGFR, estimated glomerular filtration rate; Hb, hemoglobin; HR, heart rate; IVS, interventricular septum; LA, left atrium; LDH, lactate dehydrogenase; LV, left ventricle; LVEDD, left ventricular end-diastolic diameter; LVESD, left ventricular end-systolic diameter; LVEF, left ventricular ejection fraction; LVMI, left ventricular mass index; LVmean, left ventricular mean standardized uptake value; LVmax, left ventricular maximum standardized uptake value; NT-proBNP, N-terminal fragment of pro-hormone brain natriuretic peptide; PASP, pulmonary artery systolic pressure; PWT, posterior wall thickness; RWT, relative wall thickness; SBP, systolic blood pressure; TAPSE, tricuspid annular plane systolic excursion; TR, tricuspid regurgitation.

**Table 2:** Clinical data of the 9 deceased patients.

No.	Sex	Age (year)	Diagnosis	Survival time (m)	Cause of death	LVEDD (mm)	Biplane EF%	cTnI (μg/L)	hsCRP (mg/L)	Nt-proBNP (pg/ml)	Arrhythmia	LVmax	Myocardial biopsy
1	F	29	Immune-related severe myocarditis	3	Cardiogenic shock	55	35	1.23	88	2,691	Frequent ventricular, premature beats, atrial fibrillation	3.01	NA
2	M	41	Myositis	41	Cardiogenic shock	56	48	1.14	0.46	4,220	Premature beats, intraventricular conduction block	2.27	Multifocal lymphocytic infiltration positive stain for CD3 CD4,CD8,CD20,CD68
3	F	70	SLE	12	Cardiogenic shock	45	57	0.05	1.49	635	Persistent atrial fibrillation; History of ventricular fibrillation	2.49	NA
4	F	47	Immune-related myocarditis	6	Cardiogenic shock	53	29	0.04	0.8	6,247	Premature beats, Complete right bundle branch Bbock	2.76	CD3-positive T cells per square millimeter ≥15; CD3(+), CD4(+), CD20(-), CD68(+), CD8(+)
5	F	64	Polymyositis	13	Sudden death	64	44	0.24	5.2	7,102	Persistent atrial fibrillation	2.7	No definite myocardial necrosis or inflammatory infiltration is observed
6	F	69	Dermatomyositis	18	Sudden death	69	45	0.03	40.92	2,096	Third-degree atrioventricular block; post pacemaker implantation	2.41	NA
7	F	71	Vasculitis	6	Sudden death	48	64	6.00	1.24	1,941	Normal heart rhythm	2.14	NA
8	M	38	EGPA	5	stroke	56	75	0.31	12.88	5,552	Normal heart rhythm	2.82	NA
9	M	76	Microscopic polyangiitis	0.5	Alveolar hemorrhage	51	50	134	0.13	35,000	Normal heart rhythm	1.96	NA

LVEDD, left ventricular end-diastolic diameter; Biplane EF%, biplane Simpson's ejection fraction; cTnI, cardiac troponin I; hsCRP, high-sensitivity C-reactive protein; NT-proBNP, N-terminal fragment of pro-hormone brain natriuretic peptide; SLE, systemic lupus erythematosus; EGPA, eosinophilic granulomatosis with polyangiitis; NA, not applicable.

Analysis of the clinical characteristics of the deceased group revealed higher levels of high-sensitivity troponin, N-terminal pro-brain natriuretic peptide (NT-proBNP), and heart rate at enrollment, along with lower hemoglobin levels and glomerular filtration rates compared to the surviving group. Twenty-two patients (56%) had a Biplane EF below 50%. Despite these findings, no significant differences were observed in echocardiographic parameters such as left ventricular systolic function, size, or myocardial remodeling indices, including left ventricular mass index (LVMI) and relative wall thickness (RWT), between the two groups. The deceased group also had a higher use of inotropic drugs compared to survivors (*p* < 0.05), whereas no significant differences were found in other medications, such as steroids, immunosuppressants, or biologics. Importantly, the left ventricular ^68^Ga-DOTATATE SUV values (both mean and maximum) were significantly higher in the deceased group (both *p* < 0.05; Table 1).

In the cardiovascular rehospitalization group, patients had a higher BMI and a trend toward increased left atrial size compared to those without rehospitalization. This group also showed a higher rate of anticoagulant use, while other biochemical markers and cardiac function parameters did not significantly differ. Similarly, the ^68^Ga-DOTATATE SUV values were notably elevated in the rehospitalization group compared to the non-rehospitalization group (both *p* < 0.05; Table 1).

Across all enrolled patients, peripheral blood high-sensitivity C-reactive protein (hsCRP) levels were significantly higher than the normal range. However, there were no significant differences in hsCRP levels between the mortality and survival groups, nor between those with and without cardiovascular rehospitalization(*p* > 0.05 for all comparisons).

### Association between ^68^Ga-DOTATATE PET/CT uptake and myocardial macrophage inflammation

**Figure 2:**
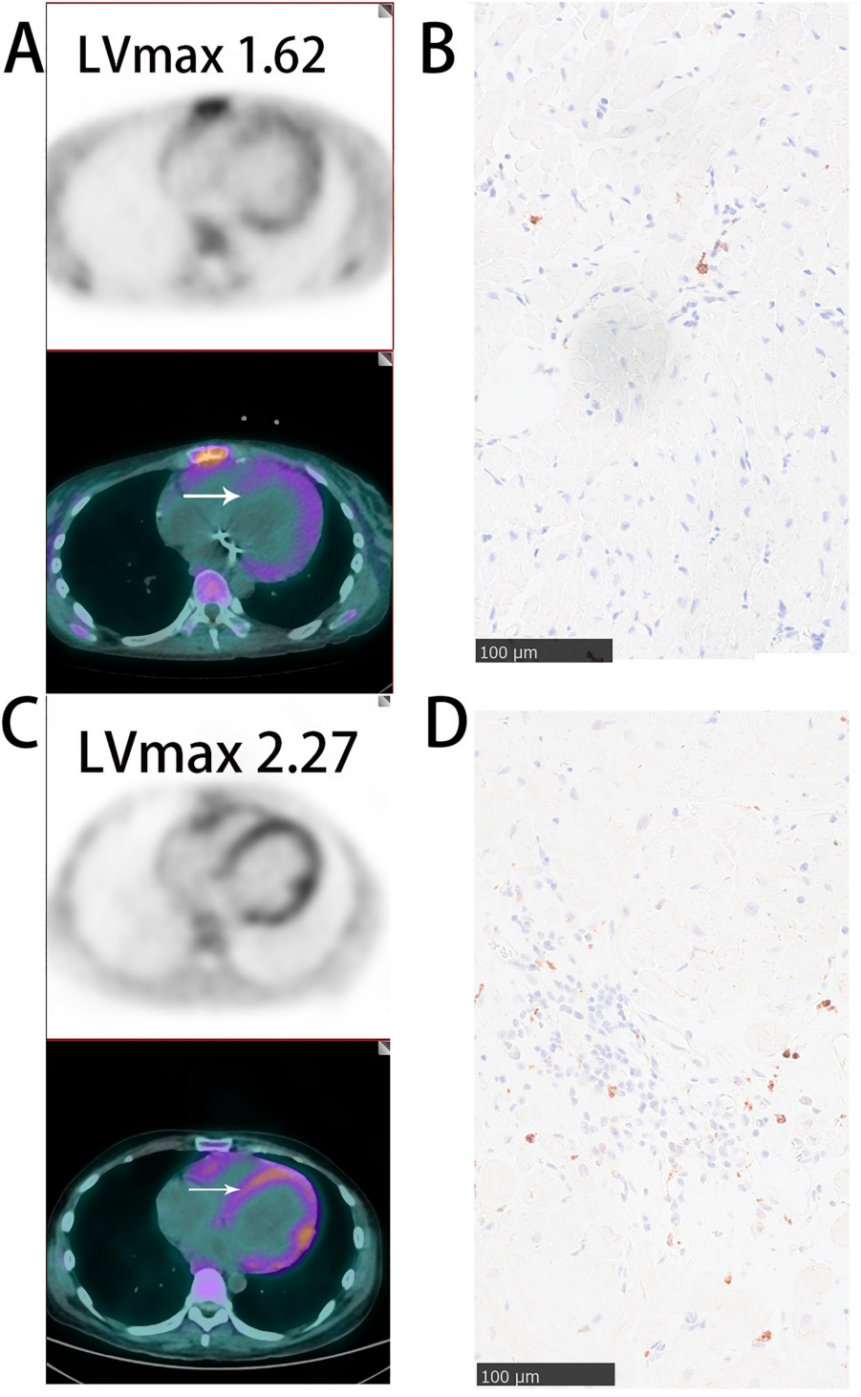
Representative images of myocardial ^68^Ga-DOTATATE PET/CT and CD68 immunohistochemical staining **(A,B)** SLE patient with mild myocardial inflammation: sparse myocardial ^68^Ga-DOTATATE uptake(lVmax = 1.62) and few CD68-positive macrophages. **(C,D)** Polymyositis patient with significant myocardial inflammation: marked myocardial ^68^Ga-DOTATATE uptake (LVmax = 2.27) and abundant CD68-positive macrophages. White arrows indicate myocardial biopsy sites. Scale bar = 100 μm.

We validated the utility of ^68^Ga-DOTATATE PET/CT myocardial imaging for detecting macrophage-related myocardial inflammation from three perspectives. First, we provided evidence showing a clear correlation between ^68^Ga-DOTATATE PET/CT myocardial imaging and the intensity of CD68-marked macrophages in myocardial biopsies (Figure 2). Second, we demonstrated the complementary diagnostic value of ^68^Ga-DOTATATE PET/CT myocardial imaging to myocardial biopsy, especially in clinically challenging cases where biopsy yield negative or uncertain results (Table 2). Finally, we separately analyzed individual clinical cases of Takayasu arteritis, demonstrating good consistency between ^68^Ga-DOTATATE PET/CT imaging and clinical disease actively levels (details provided in a dedicated subsection below).

In Figure 2, we illustrate representative cases from our cohort, specifically one patient with polymyositis and one with SLE. A notable increase in CD68-positive monocyte-macrophages was observed in the myocardium of polymyositis patient, whereas CD68-positive cells are sparse in the myocardium of the patients with SLE. Correspondingly, myocardial ^68^Ga-DOTATATE LVmax values were higher in the polymyositis case (LVmax: 2.27) compared to the SLE case (LVmax: 1.62). This finding demonstrates the ability of ^68^Ga-DOTATATE imaging to sensitively and specifically reflect monocyte-macrophages infiltration, supporting its reliability as a non-invasive method for assessing myocardial inflammation in autoimmune cardiac involvement.

### Complementary value of ^68^Ga-DOTATATE PET/CT myocardial imaging relative to myocardial biopsy

Among the enrolled patients, seven underwent myocardial biopsy. Two patients had pathological confirmation of active inflammation, including focal lymphocytic infiltration and notable CD68-positive macrophages infiltration. Their corresponding LVmax values were high at 2.76 and 2.27 respectively (Table 2: patients 4 and 2, Figure 2C). Clinically, these two patients eventually succumbed to cardiogenic shock.

Two other patients had myocardial biopsies showing no or minimal inflammatory cell infiltration (minimal CD68-positive macrophages), and their corresponding LVmax values were relatively low (LVmax at 1.7 and 1.62 Figure 2A). Both of these patients remained clinically stable throughout the follow-up period.

The remaining three patients showed no significant inflammatory response or only scattered CD3-positive lymphocytes in myocardial biopsies, yet presented notably elevated LVmax values (2.7, 2.45, and 2.40). Among them, the patient with the highest LVmax value (2.7; fifth deceased patient listed in Table 2) had negative biopsy results but demonstrated clear signs of myocardial inflammation on cardiac MRI, including T2-weighted hyperintensity in the anterolateral papillary muscle, mid-layer of the interventricular septum, and subendocardial myocardium of the left ventricular anterior and lateral walls. This patient ultimately died from cardiogenic shock. The other two patients remained stable and free of adverse clinical events during follow-up.

These findings suggest that when myocardial pathology confirms active inflammation, ^68^Ga-DOTATATE imaging aligns well with biopsy results. More importantly, in clinical challenging scenarios where myocardial biopsy results may be negative or inconclusive-possible due to sampling error or the focal nature of inflammatory infiltration- ^68^Ga-DOTATATE imaging demonstrates considerable complementary value by identifying patients with significant myocardial inflammation and elevated clinical risk.

### Predictive value of ^68^Ga-DOTATATE uptake

**Figure 3:**
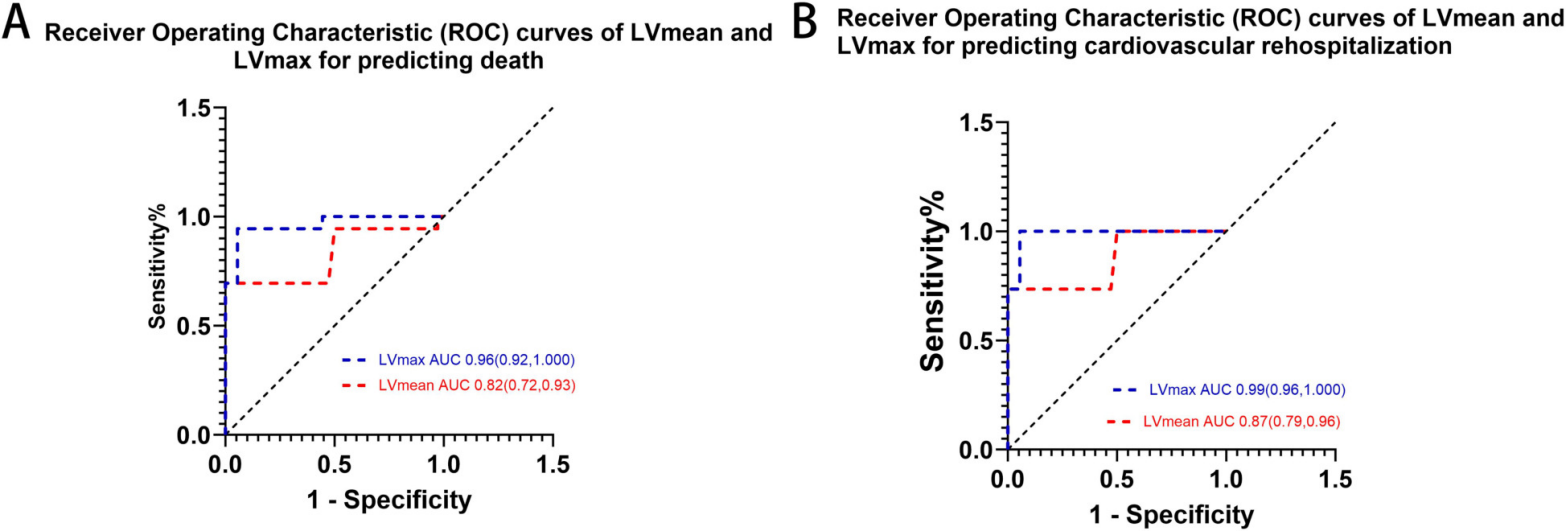
ROC curves of myocardial lVmax and lVmean and in predicting **(A)** death and **(B)** cardiovascular rehospitalization. LVmax showed superior predictive performance compared to LVmean for both outcomes.

ROC curve analysis confirmed that elevated myocardial ^68^Ga-DOTATATE uptake (LVmax and LVmean) significantly predicted clinical outcomes (mortality and cardiovascular rehospitalization) in patients with cardiac involvement of autoimmune diseases. Given the significant side effects associated with immunosuppressive and steroid treatments in autoimmune diseases, we intentionally selected thresholds that favored higher specificity, despite slightly lower sensitivity, to minimize overtreatment risk. Optimal thresholds determined from ROC analyses are provided in Figure 3 and Table 3.

**Table 3:** Prognostic value of myocardial ^68^Ga-DOTATATE uptake and clinical biomarkers.

Outcome	Marker	ROC *P*-value	AUC (95%CI)	Cut-off	Sensitivity (%)	Specificity (%)	K-M *P*-value
Death	LVmax	<0.0001	0.96 (0.92–1.00)	≥2.405	67.0	89.0	<0.0001
	LVmean	<0.0001	0.82 (0.72–0.93)	≥1.27	55.6	88.0	<0.0001
	Biplane EF	0.4	0.59 (0.38–0.79)	N/A	N/A	N/A	N/A
	NT-proBNP	0.04	0.74 (0.58–0.91)	≥1,632	88	68	0.007
	hsCRP	0.81	0.53 (0.30–0.75)	N/A	N/A	N/A	N/A
	cTnI	0.05	0.72 (0.55,0.89)	≥0.044	89	58	0.012
Cardiovascular rehospitalization	LVmax	<0.0001	0.99 (0.96–1.00)	≥2.405	55.6	84.0	<0.0001
	LVmean	<0.0001	0.87 (0.79–0.96)	≥1.36	55.6	96.0	<0.0001
	Biplane EF	0.95	0.493 (0.28,0.71)	N/A	N/A	N/A	N/A
	NT-proBNP	0.30	0.62 (0.41,0.83)	N/A	N/A	N/A	N/A
	hsCRP	0.31	0.38 (0.14,0.63)	N/A	N/A	N/A	N/A
	cTnI	0.95	0.49 (0.28,0.71)	N/A	N/A	N/A	N/A

AUC, area under the curve; Biplane EF%: Biplane Simpson's Ejection Fraction; Cl, confidence interval; cTnI, cardiac troponin I; hsCRP, high-sensitivity C-reactive protein; NT-proBNP, N-terminal fragment of pro-hormone brain natriuretic peptide.

**Figure 4:**
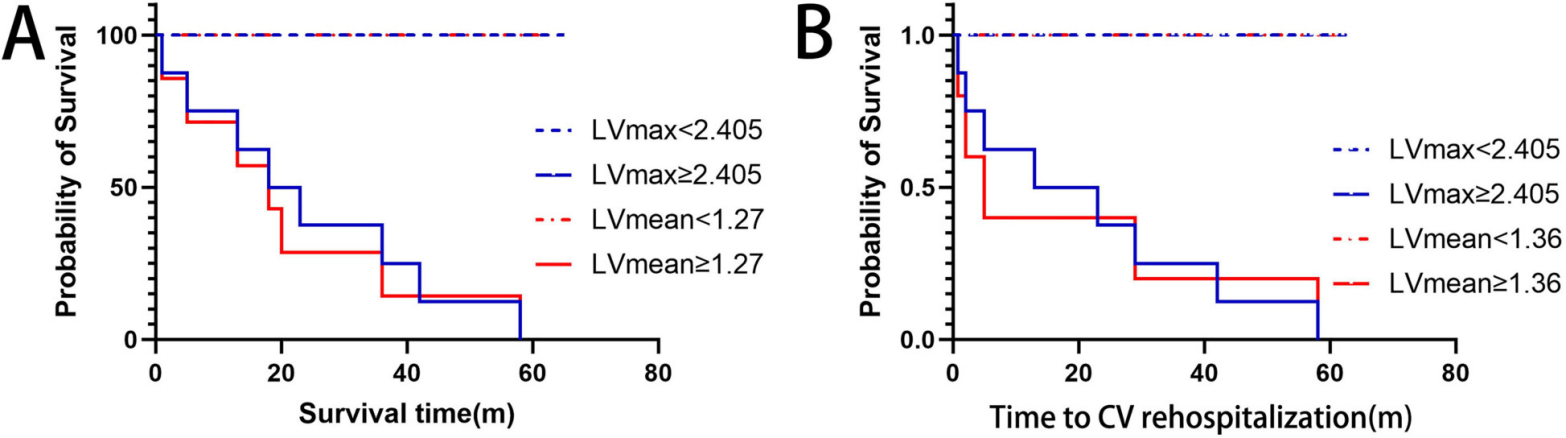
Kaplan–Meier curves for **(A)** overall survival and **(B)** cardiovascular rehospitalization, stratified by optimal lVmax and lVmean thresholds. Higher LVmax (≥2.405) and LVmean (≥1.27 for survival; ≥1.36 for rehospitalization) significantly correlate with worse outcomes.

To clearly illustrate the superiority of ^68^Ga-DOTATATE uptake parameters (LVmax and LVmean) over conventional clinical markers, we provided a comprehensive comparison table (Table 3) that includes LVmax, LVmean, NT-proBNP, EF, and cardiac troponin I (cTnI). Kaplan–Meier survival analysis using ROC-derived thresholds revealed significant differences in patient outcomes (Figure 4). Regarding all-cause mortality, patients with LVmax ≥ 2.405 exhibited significantly shorter survival compared to shoes with LVmax < 2.405(log-rank *χ*² = 38.73, *p* < 0.0001). Similarly, LVmean ≥ 1.27 effectively differentiated patient survival, with significantly worse outcomes in the higher LVmean group (log-rank *χ*² = 39.79, *p* < 0.0001).

For cardiovascular rehospitalization, similar significant differences were observed. Patients with LVmax ≥ 2.405 had significantly earlier rehospitalization compared to those with LVmax < 2.405 (log-rank *χ*² = 37.40, *p* < 0.0001). Likewise, LVmean ≥ 1.36 was associated with significantly earlier cardiovascular rehospitalization events (log-rank *χ*² = 35.63, *p* < 0.0001). Median rehospitalization-free intervals were notably shorter for patients above these thresholds, whereas patients below these thresholds generally remained free from cardiovascular rehospitalization for the duration of follow-up.

Interestingly, NT-proBNP and cardiac troponin I (cTnI) also showed significant predictive value for mortality based on Kaplan–Meier analysis (*p* = 0.007 and *p* = 0.012, respectively), but their ROC curves exhibited relatively lower specificity and AUC values, indicating inferior discriminatory power compared to LVmax and LVmean. Additionally, multivariate logistic regression analysis further supported the superiority of LVmax and LVmean as independent predictors for adverse outcomes (Supplementary Table S1). Other indicators, including biplane ejection fraction (Biplane EF) and high-sensitivity C-reactive protein (hsCRP), showed neither significant ROC curve results nor Kaplan–Meier analysis significance, confirming their limited prognostic utility in this study.

In summary, increased myocardial ^68^Ga-DOTATATE uptake, represented by LVmax and LVmean above identified thresholds, was significantly associated with adverse clinical outcomes, clearly demonstrating its superior prognostic value for mortality and cardiovascular rehospitalization.

### Examples from Takayasu arteritis: ^68^Ga-DOTATATE differentiates degrees of myocardial involvement in autoimmune disease

**Figure 5:**
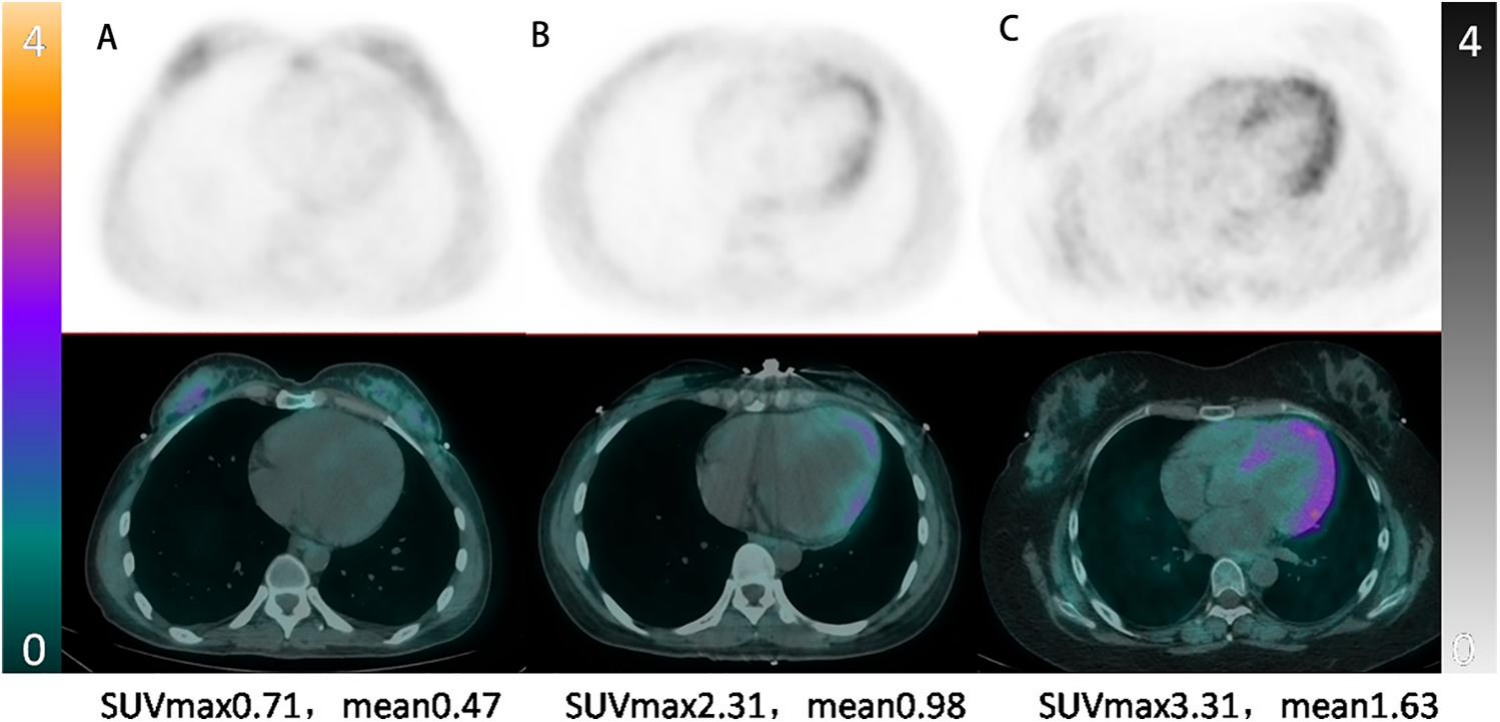
Representative myocardial ^68^Ga-DOTATATE PET/CT images illustrating varying inflammation severity in takayasu arteritis. From left to right: **(A)** 36-year-old woman, stable phase, minimal myocardial uptake (LVmax = 0.71); **(B)** 26-year-old woman, moderate myocardial inflammation post-aortic pseudoaneurysm repair (LVmax = 2.31); **(C)** 28-year-old woman, severe active myocardial inflammation requiring targeted therapy (LVmax = 3.31).

We further illustrate the clinical relevance of ^68^Ga-DOTATATE PET/CT in identifying varying degrees of myocardial involvement in Takayasu arteritis through the following representative cases (Figure 5). Patient A, a 36-year-old woman, had been living with Takayasu arteritis for over 10 years. Despite having moderate aortic regurgitation, her condition had remained stable, and she had successfully carried a pregnancy to full term. Her myocardial ^68^Ga-DOTATATE PET/CT scan showed minimal inflammation (LVmax = 0.71).

Patient B, a 26-year-old woman, was diagnosed with Takayasu arteritis three years prior and had undergone aortic valve replacement. She presented with back pain and decreased cardiac function (EF = 51%) due to a pseudoaneurysm involving the left main coronary artery, proximal to mid-LAD, and circumflex artery. Following successful pseudoaneurysm repair and anti-remodeling therapy, her condition stabilized, with myocardial ^68^Ga-DOTATATE PET/CT scan showing intermediate myocardial inflammation (LVmax = 2.31).

Patient C, a 28-year-old woman, was diagnosed with extensive Takayasu arteritis involving the aortic root (severe aortic regurgitation and aortic sinus dilation) and head and arm vessels (severe stenosis). She presented with heart failure and underwent a Bentall procedure with partial arch replacement. Due to persistent severe myocardial involvement, targeted anti-inflammatory therapy with tocilizumab was initiated, resulting in clinical improvement (EF increased from 46% to 63%). Her myocardial ^68^Ga-DOTATATE PET/CT scan confirmed severe myocardial inflammation (LVmax = 3.31).

These clinical examples underscore the sensitivity of ^68^Ga-DOTATATE PET/CT in differentiating the extent of myocardial inflammation in Takayasu arteritis, demonstrating its clinical value in guiding personalized therapeutic approaches and prognosis assessment.

## Discussion

This prospective single-center study confirms that confirms that myocardial macrophage inflammation assessed using ^68^Ga-DOTATATE PET/CT is independently associated with adverse clinical outcomes (all-cause mortality and cardiovascular rehospitalization) in patients with autoimmune-related cardiac involvement. ^68^Ga-DOTATATE PET/CT represents a feasible, safe, non-invasive, and quantitative approach for evaluating myocardial inflammation, with the potential to guide therapeutic decisions and improve prognosis in clinical practice.

This study revealed several clinically valuable findings.

### Clinical relevance of chronic macrophage inflammation

Chronic macrophage inflammation is a key predictor of poor prognosis in autoimmune diseases, as it leads to significant myocardial damage and fibrosis. This inflammation is mediated by activated macrophages that release pro-inflammatory cytokines such as TNF-α, IL-1β, and IL-6, promoting fibrosis and cardiac dysfunction (28). Additionally, these macrophages generate reactive oxygen species (ROS), leading to oxidative stress and further myocardial injury (29, 30). In autoimmune diseases, macrophages present cardiac antigens to T cells, triggering the production of autoantibodies that directly damage cardiomyocytes (10). This cascade of events leads to both diastolic and systolic dysfunction, arrhythmias, and fibrotic remodeling, all of which are key indicators of poor outcomes in heart failure. Although a strong link between macrophage infiltration and poor outcomes in autoimmune diseases has been established (31, 32), prospective studies specifically addressing cardiac involvement in autoimmune diseases are still limited. Our study adds valuable prospective evidence linking chronic macrophage inflammation with adverse cardiac prognosis in autoimmune diseases, highlighting the importance of further larger-scale studies to refine clinical strategies and treatment guidance.

### Limitations of current methods and complementary value of ^68^Ga-DOTATATE PET/CT

Peripheral biomarkers such as cardiac troponin and high-sensitivity CRP often fail to adequately reflect myocardial inflammation severity. While echocardiography is essential for diagnosing myocardial involvement, cardiac function can be influenced by various factors. In addition to myocardial inflammation, factors such as stress, fluid overload, coronary artery disease, valvular involvement, and arrhythmias can significantly impact cardiac function. Moreover, cardiac function may significantly fluctuate with appropriate treatment, thus limiting its reliability as a stable indicator of inflammation severity. This variability likely explains why traditional echocardiographic parameters and remodeling indices (e.g., LVMI, RWT) did not show significant correlations with clinical prognosis in our study. Myocardial biopsy remains the gold standard for diagnosing myocardial inflammation but has significant drawbacks including invasiveness, potential sampling errors due to the focal and heterogeneous nature of inflammatory lesions, and limited feasibility in chronic conditions (33). Typically, biopsies are performed from the interventricular septum of the right ventricle, an area that might not represent inflammation occurring predominantly in the left ventricle, potentially leading to false-negative or inconclusive results.

Cardiac MRI, despite its diagnostic value, is constrained by factors such as high costs, limited availability, renal function contraindications for contrast use, prolonged scan duration, and inability to directly quantify myocardial inflammation (34–36).

Currently, ^18^F-FDG PET remains widely used to assess myocardial inflammation (37), but interpretation challenges due to physiological myocardial glucose uptake often limit specificity (38).

In contrast, our findings highlight the complementary diagnostic utility of ^68^Ga-DOTATATE PET/CT imaging. This modality specifically targets somatostatin receptor subtype-2 (SSTR2), which is expressed by activated M1 macrophages, offering higher specificity and fewer interpretative challenges compared to ^18^F-FDG PET. Crucially, ^68^Ga-DOTATATE PET/CT is particularly valuable when myocardial biopsy results are negative or uncertain, providing a non-invasive alternative that supports critical clinical decisions, especially regarding the initiation or intensification of immunosuppressive therapy. By identifying significant myocardial inflammation and minimizing overtreatment risks through judicious threshold selection, ^68^Ga-DOTATATE PET/CT emerges as a robust and clinically relevant tool, complementing existing diagnostic approaches. Future studies directly comparing ^68^Ga-DOTATATE with other imaging methods will further define its role and potential advantages in clinical practice.

### SUV versus target-to-background ratio(TBR) in myocardial ^68^Ga-DOTATATE uptake reporting

The results of our study suggest that the left ventricular SUV value, whether maximum or mean, is more strongly correlated with prognosis than TBR. One reason for this could be that TATE primarily reflects the infiltration of M1-type mononuclear macrophages, which are unevenly distributed in the myocardium depending on the disease's etiology, three-dimensional distribution, and functional state (39). The maximum SUV value in the left ventricle is likely to represent the most severe stage of immune-mediated myocardial injury at the time of the scan, as patients—despite receiving standard treatment—still show elevated myocardial uptake, indicating that the current therapies may not be fully effective. In contrast, most patients in our study were in a stable phase of chronic macrophage-mediated inflammation, leading to relatively low SUV values. When SUV values are divided by blood pool values, the resulting TBR ratio becomes exaggerated, potentially reducing its sensitivity in accurately reflecting the disease severity. This may explain why TBR did not show a positive correlation with prognosis in our study. Furthermore, the relatively small sample size could also be a contributing factor.

### Limitations and future directions

Our study has several important limitations. First, our analyses involved multiple statistical tests without formal adjustments for multiple comparisons, potentially inflating the Type I error risk. And the relatively small sample size prevented robust subgroup analyses and formal inclusion of multivariate regression results (provided in Supplementary Table S1). Although exploratory analyses indicate promising predictive value of myocardial ^68^Ga-DOTATATE uptake parameters (LVmax and LVmean), these findings should be cautiously interpreted and require validation in larger, multicenter studies. Additionally, the heterogeneity of autoimmune conditions included limits the generalizability of our results to specific disease entities; larger, more homogeneous patient cohorts are needed to refine disease-specific prognostic thresholds. Lastly, broader clinical implementation of ^68^Ga-DOTATATE PET/CT remains constrained by higher costs, the necessity for specialized nuclear medicine infrastructure, and limited accessibility in certain regions, particularly in resource-constrained settings. Future studies should systematically address these challenges through prospective cost-effectiveness analyses, standardized imaging and reporting protocols, and evaluation of long-term clinical practicality across diverse healthcare settings.

## Conclusion

Myocardial ^68^Ga-DOTATATE uptake independently predicts adverse clinical outcomes in autoimmune-related cardiac involvement, highlighting its potential value as a non-invasive marker for myocardial inflammation. Larger multicenter studies and standardized protocols are warranted to confirm these findings and facilitate clinical implementation.

## Data Availability

The datasets presented in this article are not readily available because at this stage, our patient cohort is still under active follow-up, and the data collection is ongoing. Therefore, we are currently unable to share the original data upon external request. Once the follow-up period concludes and analyses are finalized, we will consider reasonable requests for data sharing. Requests to access the datasets should be directed to linxuepumnch@qq.com.
