# Supplementary material for: Prognostic value of 68Ga-DOTATATE PET/CT in assessing cardiac involvement in autoimmune diseases: a prospective study
Source: Front Cardiovasc Med. 2025 Oct 10;12:1598638. doi: 10.3389/fcvm.2025.1598638 (PMC12549676; doi:10.3389/fcvm.2025.1598638)
Supplement: Supplementary file 1 [file Datasheet1.pdf]

Multivariate Regression Analysis

In conducting multivariate regression analysis, we prioritized assessing the relationship between ^{68}Ga-DOTATATE uptake and prognosis rather than focusing on time-to-event analysis, given the relatively small sample size. Consequently, logistic regression analysis was used instead of Cox regression. Supplementary Table 1 presents the results of the univariate and binary logistic regression analyses used in the multivariate prognosis analysis.

Variables included in the binary logistic regression analysis were those with significant results in the univariate analysis. Although baseline cTnI levels were significantly higher in the mortality group compared to the survival group, they were not associated with outcomes in the univariate logistic regression analysis ($p = 0.230$) and were therefore excluded from the multivariate analysis. NT-proBNP levels were log-transformed during the analysis. The use of inotropic agents was higher in the deceased group, but the small number of patients receiving inotropic therapy rendered insufficient data to support inclusion in the logistic regression model. In the analysis of cardiovascular rehospitalization, biplane EF was also included.

The results of the univariate analysis were consistent with those In Table 1, showing no significant differences in left ventricular size, ejection fraction, troponin levels, or peripheral blood CRP levels between the mortality and cardiovascular rehospitalization groups.

For both mortality and cardiovascular rehospitalization, high myocardial 68Ga-DOTATATE uptake was identified as an independent risk factor. In the analysis of risk factors related to mortality, all models indicated that LVmax or LVmean were independent risk factors for death. Considering model fit, we determined that model 3, with the highest Nagelkerke R Square (0.61) and lower Akaike Information Criterion (AIC) and Bayesian Information Criterion (BIC) values, was the best fit model. In this model, an LVmax > 2.405 was associated with an OR of 25.38 (1.74, 369.48), P = 0.018. For cardiovascular rehospitalization, model 1 provided the best fit, with a Nagelkerke R Square of 0.522. In this model, a mean SUV of LV > 1.36 was associated with an OR of 29.34 (1.84, 467.72), P = 0.017.

Supplementary Table 1. Univariate and Multivariate Logistic Regression Analysis for Death and Cardiovascular Rehospitalization

**Death**	Univariable		Multivariable							
Predictors			Model 1		Model 2		Model 3		Model 4	
	OR (95% Cl)	P Value	OR (95% Cl)	P Value	OR (95% Cl)	P Value	OR (95% Cl)	P Value	OR (95% Cl)	P Value
**myocardial ^{68}Ga-DOTATATE LVmean＞1.27**	9.17(1.54,54.59)	0.015	**8.38(0.93,75.15)**	**0.058**	18.50(1.92,178.43)	0.012				
**myocardial ^{68}Ga-DOTATATE LVmax＞2.405**	14.67(2.34,92.10)	0.004					**25.38(1.74,369.48)**	**0.018**	**30.30(2.54,360.98)**	**0.007**
HR(bpm)	1.07(1.00,1.15)	0.043	1.06(0.97,1.16)	0.193			**1.10(0.99,1.22)**	**0.074**		
Lg(NT-proBNP (pg/ml)	4.89(1.00,23.85)	0.05	1.93(0.32,16.841)	0.475			0.97(0.93,1.00)	0.044		
Hb (g/L)	0.96(0.93,1.001)	0.055			0.97(0.91,1.03)	0.363			0.96(0.90,1.02)	0.21
eGFR (ml/min/1.73m^2)	0.977(0.96,1.00)	0.05			0.98(0.94,1.01)	0.225			0.98(0.95,1.02)	0.34
Nagelkerke R Square			0.46		0.48		**0.61**		0.55	
AIC			32.11		36.91		**28.67**		33.80	
BIC			34.91		39.97		**31.47**		36.86	

**Cardiovascular rehospitalization**	**Univariable**		**Multivariable**							
Predictors			Model 1		Model 2		Model 3		Model 4	
	OR (95% Cl)	P Value	OR (95% Cl)	P Value	OR (95% Cl)	P Value	OR (95% Cl)	P Value	OR (95% Cl)	P Value
myocardial 68Ga-DOTATATE LVmean＞1.36	30(2.74,328.64)	0.005	**29.34（1.84，467.72）**	**0.017**	27.34（2.16，346.25）	0.011				
myocardial 68Ga-DOTATATE LVmax＞2.405	6.56(1.21,35.73)	0.030					4.89（0.66，36.03）	0.120	8.10（1.16，56.77）	0.035
BMI(kg/m2)	1.26(1.01,1.59)	0.046	**1.17（0.90，1.53）**	**0.245**			1.23（0.97，1.57）	0.088		
LA(mm)	1.14(0.99,1.31)	0.066	**1.12（0.93,1.35）**	**0.240**			1.04（0.89，1.23）	0.600		
Biplane EF%	0.98(0.92,1.03)	0.379			0.99（0.91,1.07）	0.759			0.98（0.91，1.05）	0.59
Anti-coagulation	6.56(1.21,35.73)	0.030			5.67（0.67，47.73）	0.111			7.16（1.03，49.65）	0.05
Nagelkerke R Square			**0.52**		0.50		0.35		0.38	
AIC			**31.39**		29.60		37.23		35.58	
BIC			**34.26**		32.65		40.10		38.63	

AIC, Akaike Information Criterion; BIC, Bayesian Information Criterion; Biplane EF%, Biplane Simpson's ejection fraction; BMI, body mass index; eGFR, estimated glomerular filtration rate; Hb, hemoglobin; LA, left atrium; NT-proBNP, N-terminal pro-brain natriuretic peptide.

Supplementary Table 2 : Clinical Characteristics and Pathological Findings of All Enrolled Patients

Disease Category	Gender	Age	Outcome	hsCRP (mg/L)	cTnI (μg/L)	NT-proBNP (pg/mL)	Biplane EF%	LVmax	LVmean	Major treatment and Immunosuppressants	Myocardial Pathology
Autoimmune Myocarditis	F	47	Rehospitalization/Death	0.80	0.04	6247	29	2.76	1.65	HCQ	
Autoimmune Myocarditis	F	29	Rehospitalization/Death	88	1.23	2691	35	3.01	1.38	MTX→LEF	NA
Autoimmune Myocarditis	M	31	NA	2.87	0.14	192	43	1.52	0.74	LEF	NA
Autoimmune Myocarditis	M	65	NA	0.78	0.34	91	55	2.40	1.34	Tripterygium	NA
Behçet's Disease	M	29	NA	55.25	0.00	688	70	2.14	1.14	AZA	NA
Behçet's Disease	M	39	NA	16.80	0.02	84	70	1.22	0.64	CTX, LEF	NA
Behçet's Disease	M	27	NA	59.14	0.02	91	73	1.22	0.63	CTX→LEF	NA
Behçet's Disease	M	64	NA	4.31	0.05	6966	35	2.08	1.04	LEF	NA
CTD	F	65	Rehospitalization	1.05	0.02	489	50	1.84	0.96	MTX→Tripterygium	
CTD	F	26	Lost to follow up	0.10	11.19	16048	23	1.70	0.95	MMF	Focal cardiomyocyte hypertrophy, no inflammation
Myositis,DM	F	69	Rehospitalization/Death	40.92	0.03	2096	45	2.41	1.42	MTX+CsA	
Myositis,PM	F	44		7.20	0.02	315	46	2.09	1.25	MTX	
Myositis,PM	F	30		6.37	0.13	3741	25	1.82	1.00	MTX	

Myositis,PM	F	64	Death	5.20	0.24	7102	44	2.70	1.29	MTX→CsA	No definite myocardial necrosis or inflammatory infiltration is observed
Myositis,PM	M	54	Lost	0.67	0.10	213	64	1.39	0.77	MTX+CTX	
Myositis,PM	M	67		12.18	0.10	469	53	2.26	1.25	TAC	
Myositis,PM	M	41	Rehospitalization/Death	0.46	1.14	4220	48	2.27	1.17	MTX+CsA	Multifocal lymphocytic infiltration positive stain for CD3 CD4,CD8,CD20,CD68
Myositis,PM	M	42		1.51	1532	196	48	2.45	1.20	NA	Degenerative changes, scattered CD3+ lymphocytes
Scleroderma	F	44		0.64	NA	1324	37	1.89	0.93	MTX→LEF	
Scleroderma	M	35		1.07	0.25	NA	59	1.41	0.82	CTX	
Sjögren's Syndrome	F	72	Rehospitalization	0.97	0.03	5497	25	2.30	1.13	HCQ	
SLE	F	41		17.29	0.00	16623	34	4.11	2.22	HCQ+MMF	
SLE	F	21		0.97	0.02	1034	36	0.99	0.52	MMF+HCQ	
SLE	F	70	Death	1.49	0.05	635	57	2.49	1.21	HCQ+LEF	
SLE	F	28		0.07	13.96	3539	29	1.62	0.81	TAC+HCQ	Degenerative changes, sparse mononuclear infiltration
Systemic Vasculitis	F	71	Death	1.24	0.05	1941	64	2.14	1.13	CTX	

Systemic Vasculitis	M	47	Rehospitalization	0.30	0.00	170	38	2.01	1.03	CTX	
Systemic Vasculitis	M	54		21.92	0.02	3597	42	1.84	0.97	CTX	
Systemic Vasculitis	M	33		0.41	0.04	404	41	2.01	0.95	无	
Systemic Vasculitis	M	21		35.33	1.16	11489	71	1.49	0.88	CTX	
Systemic Vasculitis,EGPA	F	27	Rehospitalizatioin	4.20	0.00	344	27	1.91	0.95	CTX+MTX	
Systemic Vasculitis,EGPA	M	38	Rehospitalization/Death	12.87	0.31	5552	75	2.82	1.58	MMF	
Systemic Vasculitis,MPA	M	76	Death	0.13	134	35000	50	1.96	0.96	CTX	
Systemic Vasculitis,TA	F	36		2.02	0.00	NA	70	0.71	0.47	HCQ → AZA，Stable, no surgical intervention	
Systemic Vasculitis,TA	F	28	Rehospitalization	0.00	0.01	562	46	3.31	1.63	Bentall procedure and partial arch replacement, heart function improved after tocilizumab therapy.	Lymphocytic aggregates, elastic fiber disruption
Systemic Vasculitis,TA	F	26		53.24	0.03	1170	34	2.31	0.93	Aortic valve replacement and pseudoaneurysm repair, stable post-surgery and on MTX	Chronic inflammation, multifocal lymphocytes

Abbreviations: hsCRP, high-sensitivity C-reactive protein; cTnI, cardiac troponin I; NT-proBNP, N-terminal pro-B-type natriuretic peptide; eGFR, estimated glomerular filtration rate; Biplane EF%, biplane Simpson's ejection fraction; LVmax, maximum standardized uptake value; LVmean, mean standardized uptake value; HCQ, hydroxychloroquine; MTX, methotrexate; LEF, leflunomide; AZA, azathioprine; CTX, cyclophosphamide; CsA, cyclosporine A; MMF, mycophenolate mofetil; TAC, tacrolimus; TCZ, tocilizumab; NA, not applicable.
