# Supplementary figures and images for: Prognostic value of 68Ga-DOTATATE PET/CT in assessing cardiac involvement in autoimmune diseases: a prospective study
Source: Front Cardiovasc Med. 2025 Oct 10;12:1598638. doi: 10.3389/fcvm.2025.1598638 (PMC12549676; doi:10.3389/fcvm.2025.1598638)

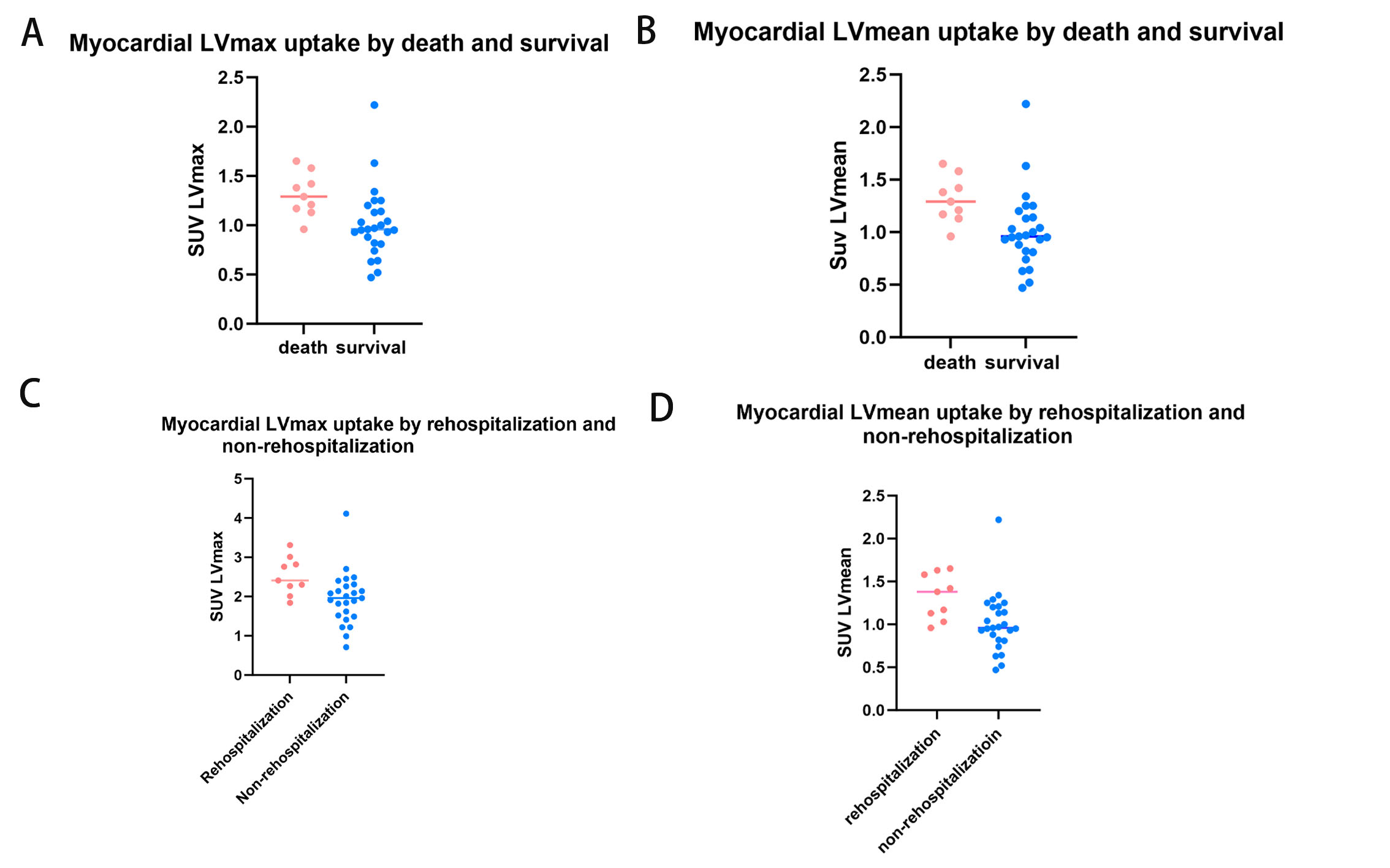

Supplement: Supplementary file 2 [file Image1.jpeg]
